# Evaluating the Test–Retest Reliability of Five Low-Cost, Perturbation-Based Functional Tests for Balance Recovery in Older Adults

**DOI:** 10.3390/sports13110375

**Published:** 2025-11-03

**Authors:** Maria Melo-Alonso, Juan Luis Leon-Llamas, Santos Villafaina, Juan Pedro Fuentes-García, Francisco Javier Domínguez-Muñoz, Narcis Gusi

**Affiliations:** 1 Grupo de Investigación en Actividad Física, Calidad de Vida y Salud (AFYCAV), Didáctica de la Expresión Musical, Plástica y Corporal, Facultad de Ciencias del Deporte, Universidad de Extremadura, Avenida de la Universidad s/n, 10003 Cáceres, Spain; leonllamas@unex.es (J.L.L.-L.); svillafaina@unex.es (S.V.); jpfuent@unex.es (J.P.F.-G.); fjdominguez@unex.es (F.J.D.-M.); 2 Instituto de Investigación e Innovación en Deporte (INIDE), 10003 Cáceres, Spain; 3 Grupo de Investigación en Análisis Didáctico y Comportamental del Deporte (ADICODE), Didáctica de la Expresión Musical, Plástica y Corporal, Facultad de Ciencias del Deporte, Universidad de Extremadura, Avenida de la Universidad s/n, 10003 Cáceres, Spain; 4 Biomedical Research Networking Center on Frailty and Healthy Aging (CIBERFES), 45071 Toledo, Spain

**Keywords:** assessment, cognitive impairment, older adults, postural control, reproducibility

## Abstract

Background: Falls are a leading cause of injury and disability among older adults. Conventional clinical tests typically do not challenge reactive postural responses to unexpected perturbations, which limits their ability to comprehensively assess fall risk. Objective: To examine the test–retest reliability of five pragmatic, low-cost, perturbation-based tests designed to identify compensatory stepping strategies in older adults, and to explore their concurrent validity against established clinical assessments. Methods: Fifty-seven older adults (44 community-dwelling and 13 institutionalized) completed five compensatory stepping tests (obstacle crossing, forward push, backward pull, and lateral pulls to the right and left) and conventional functional tests [Timed Up and Go (TUG), 30 s Chair Stand, and the Short Physical Performance Battery (SPPB)] on two separate days, ten days apart. Cohen’s weighted kappa (Kw) quantified test–retest reliability, and Pearson’s correlation coefficients assessed relationships with conventional tests. Results: Obstacle (Kw = 0.443), forward push (Kw = 0.518), and backward pull (Kw = 0.438) demonstrated moderate agreement overall. Lateral pull tests showed poor reliability. Nevertheless, moderate correlations were observed between some perturbation tests (particularly obstacle and backward pull) and standard clinical measures, especially TUG and SPPB. Conclusions: Although reliability was limited—most notably for lateral perturbations—specific tests showed meaningful associations with validated functional assessments. Pending methodological refinements, these low-cost tools may offer useful insights for initial fall-risk screening.

## 1. Introduction

The World Health Organization estimates that in 2021 there were approximately, 761 million people aged 65 years or older worldwide, a number projected to reach 1.6 billion by 2050 [1]. In Spain, 19.09% of the population already belongs to this age group [2]. With population aging, the prevalence of conditions that compromise independence and quality of life increases, with falls representing one of the most common serious threats. Approximately one in three adults over the age of 65 experiences at least one fall per year, and these events are a leading cause of injury-related morbidity, hospitalization, and mortality in this population [3]. Beyond their immediate physical consequences, such as fractures and head injuries, falls often result in long-term disability, fear of falling, reduced mobility, and loss of independence [4,5,6]. They also impose substantial economic and social burdens, making fall prevention a major public health priority [7].

Gait is a fundamental motor ability for safe mobility [8], and its deterioration is strongly associated with fall risk. Determinants such as walking speed [9], balance [10], and strength [11] have been widely studied, and various clinical tests have been developed to estimate fall risk in older adults, including the Short Physical Performance Battery (SPPB) [12], Timed Up and Go (TUG) [13], the 30 s Chair Stand [14], the Agility Challenge for the Elderly (ACE) [15], the Functional Reach Test (FRT) [16], the Single-Leg Stance Test (SLST), and the Tinetti Performance-Oriented Mobility Assessment (POMA) [16]. While these tools are inexpensive and easy to administer, they capture only partial aspects of the multifactorial nature of falls. In contrast, most falls occur in response to sudden and unpredictable disturbances, highlighting a mismatch between conventional assessments and real-world fall scenarios [17].

In recent years, perturbation-based interventions—designed to expose individuals to unexpected balance disturbances during stance or gait—have gained increasing attention. These programs aim to enhance compensatory stepping strategies, which are essential for avoiding falls or minimizing their consequences [18,19]. Evidence shows that repeated perturbation exposure improves dynamic balance and recovery responses [18,20,21,22]. Different experimental systems were applied to induce balance loss and evaluate recovery, moving-platform paradigms [18], treadmill-based slips and trips [20,21], and lateral waist-pull systems [22], all of which demonstrated improvements in gait stability, dynamic balance and reactive stepping, with some studies reporting a reduction in fall incidence [22]. Mansfield et al. [18] used a moving-platform paradigm during standing to assess compensatory stepping responses, which were found to predict fall risk in older adults. Allin et al. [20] developed treadmill-based trip and slip perturbations and performed kinematic analyses to evaluate recovery responses. Although no direct correlations with fall risk were observed, the perturbation training improved center of mass stability and enhanced proactive gait adaptations, such as step length, which may contribute to reducing fall risk. Rieger et al. [21] employed unexpected gait perturbations using a movable treadmill platform and demonstrated that perturbation training improved various spatiotemporal gait parameters, helping older adults recover balance more effectively and potentially prevent falls. Finally, Rogers et al. [22] applied repeated lateral waist-pull perturbations as both a training and assessment method, reporting that improvements in reactive stepping translated into a significant reduction in prospective fall incidence. Although repeated exposure to perturbations can improve dynamic balance [18,20,21,22], many of the tools used are technologically demanding and ill-suited to clinical practice.

However, despite these promising results, these protocols typically require high-cost, technologically demanding systems (e.g., waist-pull devices, movable platforms, release systems) to evaluate perturbation responses [23], which limits their application in routine clinical practice. Although more accessible approaches, such as the Reactive Balance Rating [24], have been developed to assess reactive balance, their use remains largely confined to specialized environments. Consequently, there is a clear methodological gap: the absence of low-cost, clinically feasible perturbation-based tests to reliably assess compensatory protective step strategies in older adults. The present study addresses this methodological issue by analyzing the test–retest reliability of five low-cost tests that are easy to administer, require minimal equipment, and are designed for use in clinical settings to identify compensatory protective stepping strategies in older adults.

## 2. Materials and Methods

### 2.1. Participants

Fifty-seven healthy older adults (women and men) were enrolled in this cross-sectional study. Recruitment constraints resulted in two groups: 44 community-dwelling participants (non-institutionalized) and 13 institutionalized participants (institutionalized).

A priori, the statistical power for Cohen’s kappa-based reliability analyses was estimated via empirical simulation in R (version 4.5.1; irr package). Assuming an expected agreement of Kappa = 0.70 (“good” reliability) versus a null hypothesis of Kappa = 0.40 at α = 0.05, we generated 1000 simulations of binary categorical data (two equally probable categories) with sample sizes matching the study groups (*n* = 44 and *n* = 14). Estimated power was 0.99 for *n* = 44 and 0.82 for *n* = 14, indicating adequate power to detect a kappa value significantly greater than the null in both groups. These results suggest sufficient power to detect substantial levels of agreement in the total sample and within subgroups.

Inclusion criteria were: (a) age ≥ 60 years; (b) ability to ambulate independently without assistance; and (c) provision of written informed consent. Exclusion criteria were: (a) psychiatric or neurological disorders; (b) substance abuse or dependence; (c) contraindications to physical exertion; (d) diseases or active/ongoing ear infections affecting balance; (e) other conditions that impair balance; and (f) serious fractures within the previous six months (e.g., hip fracture, severe ankle sprain).

The study protocol was approved by the Research Ethics Committee of the University of Extremadura (approval reference: 125/2024). All participants were informed of the procedures and provided written informed consent in accordance with the Declaration of Helsinki.

### 2.2. Instruments and Procedure

Participants reported demographic information (education, occupation) and clinical history (pharmacological therapies, diagnosed diseases, history of falls, and pain assessed using a visual analogue scale). Physical activity level was assessed with the Spanish short version of the International Physical Activity Questionnaire (IPAQ), which has demonstrated good reliability and validity in Spanish populations [25]. Cognitive status was evaluated with the Montreal Cognitive Assessment (MoCA), a multidomain screening tool covering attention, concentration, executive functions, memory, language, visuoconstruction, calculation, and orientation [26]. MoCA has greater sensitivity than the MMSE [27], with a recommended cut-off score of 23/30 to reduce false positives [28]. Health-related quality of life was assessed using the EQ-5D-5L (mobility, self-care, usual activities, pain/discomfort, anxiety/depression), including a visual analogue scale (VAS) for general health, validated in Spanish populations [29]. Fear of falling was measured using the Spanish version of the Falls Efficacy Scale-International (FES-I) [30], which has shown high reliability and validity [31,32]. Anthropometrics and age were recorded with a Tanita Body Composition Analyzer BC-418 MA (Tanita Corp., Tokyo, Japan) and a SECA 769 column scale and stadiometer (SECA Corp., Hanover, MD, USA).

**Figure 1:**
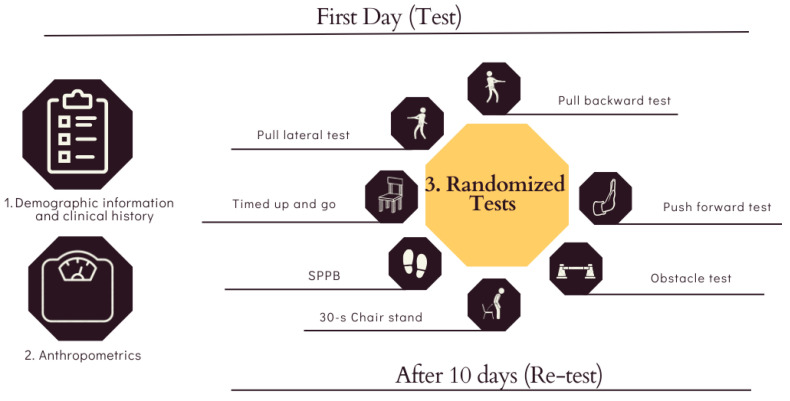
Assessment Procedure and Testing Flow.

On day 1 and again ten days later (day 2), participants completed four conventional functional tests and five compensatory stepping tests, all administered by the same evaluator on both occasions. The ten-day interval aimed to minimize learning effects [33,34]. Conventional tests were: (1) Timed Up and Go (TUG)—standing up from a chair without armrests, walking 3 m at a comfortable pace without running, turning around a cone, returning, and sitting down [35]; (2) 30 s Chair Stand—assessing lower-limb strength/power [36]. The number of repetitions was recorded, with participants instructed to perform as many repetitions as possible while maintaining correct technique; (3) SPPB—comprising standing balance tasks, 4 m usual gait speed, and a five-repetition sit-to-stand [12]; and (4) five new tests as described below in Section 2.3. Before the actual tests, participants performed one familiarization trial of TUG and a brief 30 s Chair Stand practice (three complete repetitions). A one-minute seated recovery preceded each actual test. Test order was randomized, see Figure 1.

### 2.3. Test for Compensatory Protective Step Strategies

The five tests were designed to determine compensatory protective stepping strategies through direct observation using predefined criteria. Strategy selection may be associated with fall risk, and prior work has shown that older adults more frequently adopt less efficient and less safe strategies than younger adults [23]. Scoring was informed by previous literature and anchored to the conceptual framework of the SPPB [12]. In our scheme, lower scores denote safer and more efficient strategies, whereas higher scores correspond to less effective or riskier strategies. Standardized terminology for compensatory stepping strategies was adopted based on recent Delphi-based consensus recommendations [37].

- Obstacle test: Participants walked 7.5 m at a comfortable pace. A rectangular obstacle (40 cm long × 14 cm high × 8 cm wide) was placed midway along the walkway. Scoring (1–3 points): 1 = long step strategy used effectively to clear the obstacle (safest); 2 = short step strategy used to clear the obstacle; 3 = failed execution of the chosen strategy (e.g., tripping and interrupting gait). Prior studies suggest that when reaction time permits, a lowering/long step strategy is the safer option [23,38,39]; therefore, the lowest score was assigned to the safest strategy.
- Push forward test: Standing with both feet hip-width apart, participants received a manual forward perturbation from the evaluator and were instructed to recover balance and return to the initial position. Scoring (1–2 points): 1 = single forward step longer than a usual step; 2 = multiple steps. Multiple steps to recover balance are associated with increased fall risk [40]; thus, the single-step response was scored as safer. The push was applied at a random time between 0 and 30 seconds to minimize anticipation. In addition, the evaluator applied the push form an approximately 4 cm distance to the upper back, delivered quickly, strongly and sharply.
- Pull backward test: Standing with both feet hip-width apart, participants received a manual backward perturbation (pull). Scoring (1–2 points): 1 = single backward step longer than a usual step; 2 = multiple steps. The pull was applied at a random time between 0 and 30 seconds to minimize anticipation. In addition, the evaluator performed a 4 cm pull at hip level using a rope, at maximal velocity and strongly.
- Pull lateral test (right and left): Standing with both feet hip-width apart, participants received manual lateral pulls to the right and left in random order. Scoring (1–4 points): 1 = loaded sidestep; 2 = unloaded sidestep or medial sidestep; 3 = crossover step; 4 = limb-collision compensatory step. Older adults more often exhibit unloaded or medial sidesteps, multiple steps, and collision between feet than younger adults [23,41,42]; these strategies are generally less efficient and may increase instability and collision risk [23,42]. Accordingly, the loaded sidestep received the safest score (1). Each pull was applied at a random time between 0 and 30 seconds to minimize anticipation. In addition, the evaluator performed a 4 cm pull at hip level using a rope, at maximal velocity and strongly.

All tests were video-recorded using two Xiaomi Redmi Note 8 smartphones (Xiaomi Corp., Beijing, China) positioned at 1.5 m height: one lateral view at 2.5 m and one frontal view at 4 m. Further details are provided in the Supplementary Materials (Document S1).

### 2.4. Timing and Repetition Measurement

A manual stopwatch was used during SPPB balance and 4 m gait speed tasks. An automatic Chronopic system (Chronojump, BoscoSystem^®^, Barcelona, Spain) was used for TUG via a DIN A4-sized contact platform placed on the backrest of the chair to open/close the circuit and capture test time [33,43]. The same device was placed on the seat to count repetitions during the 30 s Chair Stand and the SPPB five-times sit-to-stand.

### 2.5. Statistical Analysis

Analyses were performed using SPSS (version 25.0; IBM Corp., Armonk, NY, USA). Parametric tests were conducted after verifying normality with the Shapiro–Wilk test, and the Mann–Whitney U test was used to compare the baseline characteristics of the two independent groups (institutionalized and non-institutionalized).

Weighted Cohen’s kappa (Kw; linear and quadratic weights) [44] was used to evaluate test–retest reliability for each item, with significance set at *p* ≤ 0.05. Kw was interpreted as [45]: poor to fair (≤0.40), moderate (0.41–0.60), substantial (0.61–0.80), and excellent (≥0.81). In addition, Intraclass Correlation Coefficients (ICC) were calculated for each test by group and for the total score of each test, to enable comparisons with previous studies. Concurrent validity was explored using Pearson’s correlations between each compensatory test and TUG, 30 s Chair Stand, and SPPB scores. Following Schober [46], correlation strengths were categorized as negligible (0.00–0.10), weak (0.10–0.39), moderate (0.40–0.69), strong (0.70–0.89), and very strong (0.90–1.00). Statistical significance was set at *p* ≤ 0.05.

## 3. Results

### 3.1. Participants

Forty-four community-dwelling participants (39 women, 5 men) and thirteen institutionalized participants (3 women, 10 men) completed the study. Community-dwelling women and men had mean (SD) ages of 70.33 (4.82) and 71.80 (4.92) years, weights of 71.00 (13.05) and 76.24 (5.48) kg, and heights of 1.56 (0.09) and 1.66 (0.04) m, respectively. Institutionalized women and men had mean (SD) ages of 79.67 (8.33) and 77.60 (8.46) years, weights of 75.50 (16.68) and 78.90 (17.23) kg, and heights of 1.53 (0.03) and 1.66 (0.09) m, respectively. Both groups exhibited fear of falling and MoCA scores consistent with cognitive decline (MoCA ≤ 23), although the institutionalized group presented greater cognitive deterioration. Fear of falling was low-to-moderate among community-dwelling participants and high among institutionalized participants; see Table 1. Significant between-group differences were observed for age (*p* = 0.002), height (*p* = 0.025), MoCA (*p* = 0.001), FES-I (*p* = 0.010), TUG (*p* < 0.001), and 30 s Chair Stand (*p* < 0.001).

### 3.2. Test–Retest Reliability of Perturbations Tests

Reliability outcomes for each compensatory test are summarized in Table 2. In the total sample, obstacle (Kw = 0.443; 95% CI: 0.229–0.656), forward push (Kw = 0.518; 95% CI: 0.307–0.729), and backward pull (Kw = 0.438; 95% CI: 0.146–0.729) showed moderate agreement. Among community-dwelling participants, moderate agreement was observed for forward push (Kw = 0.495; 95% CI: 0.285–0.705) and backward pull (Kw = 0.488; 95% CI: 0.190–0.787). Among institutionalized participants, obstacle showed moderate agreement and backward pull achieved excellent agreement (Kw = 1.00; 95% CI: 1.00–1.00). Lateral pull tests exhibited poor reliability overall.

Since there were participants in the non-institutionalized group who obtained higher or lower scores on the MoCA, it was decided to conduct a detailed study of the different perturbation-based functional tests, considering the score of cognitive impairment provided by MoCA. In this sense, test–retest reliability outcomes for compensatory protective step strategies in the non-institutionalized group are summarized in Table 3. In participants with MoCA ≤ 23, push forward showed moderate agreement (Kw = 0.529; 95%CI: 0.135–0.924), whereas obstacles (kw = 0.463; 95% IC: 0.185–0.741) and backward pull (kw = 0.294; 95% IC: −0.141–0.730) yielded lower values. In those with MoCA >23, backward pull demonstrated substantial reliability (Kw = 0.704; 95% IC: 0.328–1.079), while lateral pulls to the left and right showed poor to fair agreement.

### 3.3. Correlations

Table 4 presents correlations between compensatory tests and conventional assessments. In community-dwelling participants, the obstacle test correlated significantly with TUG at retest (r = 0.448), and with SPPB at test (r = −0.443) and retest (r = −0.354). In the total sample, significant relationships were evident for most comparisons involving the obstacle test, except for the 30 s Chair Stand at test. For the backward pull test, significant values were observed in the community-dwelling group for TUG at retest (r = 0.305). In the total sample, the backward pull test correlated significantly at retest with TUG (r = 0.403), 30 s Chair Stand (r = −0.267), and SPPB (r = −0.370). For the right lateral pull, significant associations were found with SPPB at test in community-dwelling participants (r = −0.338) and, in the total sample at retest, with TUG (r = 0.332) and 30 s Chair Stand (r = −0.296).

## 4. Discussion

The aim of this study was to analyze the reliability of five pragmatic, low-cost, perturbation-based tests designed to identify compensatory protective stepping strategies in older adults. This represents a pioneering attempt to create simple, inexpensive field tests. However, the results indicate that only the forward and backward push tests demonstrated moderate agreement, with confidence intervals ranging from poor to substantial agreement. In addition, some correlations were observed between the compensatory protective stepping tests and the TUG, the 30-Second Chair Stand Test, and the SPPB. Overall, these findings suggest that the five tests do not exhibit sufficient reproducibility, as reflected by low Kappa coefficients. Nevertheless, the moderate-to-strong correlations found for the obstacle test, the backward pull test, and the right lateral pull test with the TUG, the 30-Second Chair Stand Test, and/or the SPPB support their potential convergent validity in assessing fall risk.

In the current literature, tripping simulations are commonly used. Some studies employ treadmill deceleration or acceleration methods [47], while others focus on obstacle avoidance strategies [39]. The treadmill approach is widely used to analyze the relationship between trunk kinematics and kinetics and recovery responses [38,48,49]. Although frequently applied, this method presents some inconsistencies [50], even though Shih et al. [50] proposed a reliable treadmill-based protocol. Despite this, its high cost and laboratory requirements limit its applicability in clinical settings. For these reasons, obstacle avoidance strategies were adopted, as these require only an obstacle and a walkway, making them inexpensive and easy to implement. They could serve as an initial cost-effective screening tool to detect gait problems or support clinical assessments.

The obstacle test demonstrated low reliability across all groups and in the overall sample, with Cohen’s Kappa values below acceptable thresholds. This may be due since participants were instructed to walk at their preferred pace. Variability in walking speed could have influenced compensatory gait responses and, consequently, the reliability of the test. Walking speed is a crucial factor in these assessments, as it affects the stepping strategy used during obstacle avoidance. According to Chen et al. [39], older adults are more likely to adopt a short-step strategy at both high and low walking speeds, as it is easier for them to execute compared to younger adults, who more often use the long-step strategy. The latter, however, is safer [23]. The choice of strategy depends on the time available to react to the obstacle, which is determined by walking speed: the faster the pace, the shorter the reaction time. A potential solution to this limitation would be to have participants complete the obstacle test four times—two at a comfortable walking speed and two at their maximum walking speed, presented in randomized order. This design could help identify the stepping strategies participants tend to use at different speeds, providing insight into their ability to adapt gait to environmental demands. Such demands are influenced by external conditions (e.g., outdoor obstacles or uneven surfaces) [51], as well as motor control, balance, environmental perception, and motor planning [52]. Notably, motor planning is closely linked to cognitive flexibility. Early detection of deficits in these areas may guide the development of targeted interventions to reduce fall risk in older adults.

Release and pull methods have also been used to evaluate balance recovery through compensatory stepping responses, either forward or backward. More recent protocols have attempted to increase control and replicability, particularly regarding release speed or the force applied during the pull [53,54,55,56]. However, as with the obstacle test, these methods are not easily applicable in clinical practice due to their technical complexity. In line with this study’s objectives, two simplified tests—the forward push and the backward pull—were developed, based on previously established protocols [53,54,55,56]. In these tests, the perturbation distance was set at 4 cm [41,57,58,59], and the evaluator performed both movements at maximum speed. The results of the present study demonstrated moderate reliability across the overall sample and within both institutionalized and non-institutionalized groups. Notably, among non-institutionalized participants, those without cognitive impairment showed strong reliability, whereas those with cognitive impairment exhibited lower reliability. This pattern is consistent with previous studies indicating that mild cognitive impairment compromises reactive balance control and increases response variability under both single and dual-task perturbations [60,61]. A plausible explanation is that unimpaired individuals can consistently select the same transition strategy across sessions, suggesting the learning of a specific response pattern [62,63]. Consequently, this group may benefit from shorter response times, greater motor control, and more effective motor planning. Future studies with larger and more diverse samples are warranted to further clarify the role of cognitive status in test reliability and to determine whether compensatory strategies differ across populations. This study represents the first attempt to translate a laboratory-based test into a low-cost clinical assessment. To increase standardization, the researchers designed protocols with more consistent perturbation directions and displacements (backward pull and forward push), thereby facilitating execution and identification of compensatory stepping strategies. Nevertheless, the findings cannot be directly compared to laboratory-based studies, where variables such as speed, acceleration, displacement, and perturbation type are more rigorously controlled. Moreover, laboratory perturbations are typically less predictable for participants, unlike the more structured procedures used here.

Similarly, the lateral pull test represents another attempt to standardize a laboratory-based perturbation into a low-cost clinical tool. The lateral pull test is commonly employed to analyze compensatory protective stepping responses following perturbations in the sagittal plane [41,55,64,65,66]. In this study, the evaluator applied a 4 cm pull at maximum speed, following standardized instructions. As with the push and backward pull tests, the relatively low variability in speed, acceleration, and direction may have allowed participants to anticipate perturbation during the second trial. Nevertheless, these tests may be valuable as screening tools, as the initial responses recorded in the first trial were unanticipated. This provides insight into older adults’ natural abilities to respond to sudden perturbations and helps identify innate compensatory strategies. With targeted training, such responses can be strengthened or replaced with more effective motor strategies, as demonstrated by Mansfield et al. [18] and Rogers et al. [22].

Despite these limitations, significant correlations were found between some of the new tests—particularly the obstacle test, the backward pull test, and the right lateral pull test—and established functional measures such as the Timed Up and Go (TUG) [13], the 30-Second Chair Stand Test [14], and the Short Physical Performance Battery (SPPB) [12]. These findings suggest that, although the new instruments are not yet sufficiently reliable, they may still provide valuable information regarding the motor strategies older adults adopt in response to perturbations and their relationship to fall risk. Importantly, the tests developed in this study differ from other low-cost clinical tools, such as the SPPB [12], TUG [13], 30-Second Chair Stand Test [14], Agility Challenge for the Elderly (ACE) [15], Functional Reach Test (FRT) [16], Single-Leg Stance Test (SLST), or the Tinetti Performance-Oriented Mobility Assessment (POMA) [16], as these do not involve external perturbations and therefore offer greater objectivity and reliability.

An important consideration when interpreting these findings is whether the modest reliability reflects test limitations or true variability in compensatory responses. The non-standardized walking speed in the obstacle test and the manual application of perturbations may have reduced reproducibility. At the same time, variability in compensatory protective stepping is inherent to balance recovery, which depends on motor control, environmental perception, motor planning, and cognitive flexibility. Evidence from Batcir et al. [41] or Borrelli et al. [19] shows that recurrent fallers exhibit greater variability in step initiation, step duration, larger centre of mass displacement and different strategy selection compared to non-fallers, suggesting that such differences are systematic and clinically meaningful rather than random noise. Taken together, the modest reliability observed here likely reflects a combination of methodological constraints and genuine variability in protective responses, the latter of which may offer valuable insights for individualized fall-risk assessment.

## 5. Practical and Clinical Implications

Developed perturbation-based tests, despite their limited reliability, may provide useful preliminary information for clinicians and therapists. The initial, unanticipated responses observed could help identify deficits in gait adaptability, balance recovery, and compensatory stepping strategies that are not captured by traditional functional tests. This approach may support the early detection of individuals at higher risk of falls.

In practice, these tests could be implemented in clinical or community settings as low-cost screening tools, particularly when complemented with simple modifications such as trials at different walking speeds or longer intervals between assessments. The outcomes may guide targeted interventions focused on balance training, obstacle negotiation, and cognitive-motor functions, ultimately contributing to fall prevention and the preservation of independence in older adults.

## 6. Conclusions

An initial effort to translate laboratory perturbation paradigms into low-cost, clinically applicable tests for assessing compensatory stepping strategies in older adults is presented. Reliability was limited overall—particularly for lateral perturbations—yet several tests showed moderate agreement and meaningful correlations with established functional assessments. With methodological refinements and further validation, these pragmatic tools could support affordable, field-based screening of reactive balance capacity and inform targeted preventive interventions

## Figures and Tables

**Table 1:** Descriptive characteristics of the participants by sex.

	N-I		I	
Variables	Men (*n* = 5) M (SD)	Women (*n* = 39) M (SD)	Men (*n* = 10) M (SD)	Women (*n* = 3) M (SD)
Age (years)	71.80 (4.92)	70.33 (4.82)	77.60 (8.46)	79.67 (8.33)
Height (m)	1.66 (0.04)	1.56 (0.09)	1.66 (0.09)	1.53 (0.03)
Weight (kg)	76.24 (5.48)	71 (13.05)	78.90 (17.23)	75.50 (16.68)
BMI (kg/m^2^)	27.78 (2.37)	29.86 (5.68)	28.35 (4.86)	30.70 (4.75)
Falls in the last six months	0.20 (0.45)	0.31 (0.66)	0.40 (0.52)	0.33 (0.58)
Falls in the last year	1 (2.24)	0.42 (0.86)	0.20 (0.42)	0.33 (0.58)
FES-I	18 (1.22)	22.87 (4.91)	28.36 (8.94)	28.63 (1.29)
MoCA	21.40 (3.21)	23 (5.20)	15.20 (6.70)	11.67 (7.10)
IPAQ Classification				
Intense physical activity	1 (20%)	15 (38.5%)	-	-
Moderate physical activity	4 (80%)	22 (56.4%)	7 (70%)	2 (66.7%)
Low physical activity	-	2 (5.1%)	3 (30%)	1 (33.3%)
TUG (s)	7.03 (1.68)	7.89 (1.59)	20.96 (10.66)	18.14 (4.17)
30s Chair (reps)	11.40 (1.34)	12.44 (2.56)	4.80 (1.69)	8.33 (4.04)
SPPB (Pts)	11 (0.71)	11.08 (1.11)	7 (1.25)	5.33 (1.53)

BMI = Body Mass Index; 30s Chair= chair stand test; FES-I = Fall Efficacy Scale International; I= Institutionalized; IPAQ = International Physical Activity Questionnaire; M = Mean; MoCA = Montreal Cognitive Assessment; N-I = Non-Institutionalized; SPPB= short physical performance battery; SD = standard deviation; TUG = timed up and go.

**Table 2:** Test–retest reliability of each compensatory protective step strategy test.

	I (*n* = 13)			N-I (*n* = 44)			Total (*n* = 57)		
	$k_{lw}$ (95% CI)	$k_{qw}$ (95% CI)	ICC (95% CI)	$k_{lw}$ (95% CI)	$k_{qw}$ (95% CI)	ICC (95% CI)	$k_{lw}$ (95% CI)	$k_{qw}$ (95% CI)	ICC (95% CI)
Obstacle test	0.195 (−0.254–0.644)	0.182 (−0.400–0.763)	0.183 (−0.383–0.652)	0.375 (0.142–0.609)	0.375 (0.142–0.609)	0.500 (0.242–0.692)	0.350 (0.144–0.555)	0.443 (0.229–0.656)	0.444 (0.210–0.630)
Push forward test	0.418 (−0.124–0.960)	0.418 (−0.124–0.960)	0.424 (−0.140–0.780)	0.500 (0.257–0.743)	0.495 (0.285–0.705)	0.527 (0.275–0.711)	0.518 (0.307–0.729)	0.518 (0.307–0.729)	0.539 (0.326–0.700)
Pull backward test	1 (1–1)	1 (1–1)	0.000 (−0.532–0.532)	0.488 (0.190–0.787)	0.488 (0.190–0.787)	0.500 (0.242–0.692)	0.438 (0.146–0.729)	0.438 (0.146–0.729)	0.440 (0.204–0.627)
Pull laterally left test	0.086 (−0.130–0.303)	0.185 (−0.168–0.538)	0.308 (−0.269–0.722)	0.058 (−0.064–0.179)	0.242 (0.068–0.416)	0.366 (0.081–0.596)	0.067 (−0.042–0.176)	0.239 (0.086–0.392)	0.365 (0.118–0.570)
Pull laterally right test	0.373 (−0.082–0.829)	0.241 (−0.433–0.915)	0.256 (−0.320–0.819)	0.267 (0.054–0.481)	0.270 (−0.015–0.554)	0.291 (0.003–0.539)	0.285 (0.080–0.490)	0.253 (−0.036–0.543)	0.260 (0.002–0.486)

CI = confidence interval; I = Institutionalized; ICC = intraclass correlation coefficient; $k_{lw}$ = linear weighted Kappa value; N-I = No- Institutionalized $k_{qw}$ = quadratic weighted Kappa value.

**Table 3:** Test–retest reliability of each compensatory protective step strategy test in the non-institutional group, with and without cognitive impairment.

	MoCA ≤ 23 (*n* = 20)			MoCA > 23 (*n* = 24)		
	$k_{lw}$ (95% CI)	$k_{qw}$ (95% CI)	ICC (95% CI)	$k_{lw}$ (95% CI)	$k_{qw}$ (95% CI)	ICC (95% CI)
Obstacle test	0.333 (0.001–0.665)	0.463 (0.185–0.741)	0.527 (0.122–0.781)	0.281 (−0.029–0.592)	0.378 (0.089–0.668)	0.382 (−0.016–0.676)
Push forward test	0.529 (0.135–0.924)	0.529 (0.135–0.924)	0.542 (0.143–0.790)	0.440 (0.118–0.762)	0.440 (0.118–0.762)	0.480 (0.104–0.736)
Pull backward test	0.294 (−0.141–0.730)	0.294 (−0.141–0.730)	0.301 (−0.151–0.649)	0.704 (0.328–1.079)	0.704 (0.328–1.079)	0.722 (0.456–0.869)
Pull laterally left test	0.079 (−0.032–0.190)	0.183 (−0.056–0.423)	0.331 (−0.119–0.668)	0.051 (−0.143–0.244)	0.298 (0.074–0.522)	0.400 (0.005–0.687)
Pull laterally right test	0.237 (−0.041–0.515)	0.191 (−0.166–0.548)	0.231 (−0.224–0.604)	0.269 (−0.026–0.562)	0.345 (0.000–0.690)	0.349 (−0.054–0.655)

CI = confidence interval; I = Institutionalized; ICC= intraclass correlation coefficient; $k_{lw}$ = linear weighted Kappa value; MoCA = Montreal Cognitive Assessment; N-I = No- Institutionalized $k_{qw}$ = quadratic weighted Kappa value.

**Table 4:** Correlations between the value obtained in each compensatory protective step strategy test in test and retest with TUG, 30 s Chair and SPPB.

	Obstacle Test			Push Forward Test			Pull Backward Test			Pull Laterally Left Test			Pull Laterally Right Test		
Variables	I (*n* = 13) *p*-Value	N-I (*n* = 44) *p*-Value	Total (n = 57) *p*-Value	I (n = 13) *p*-Value	N-I (*n* = 44) *p*-Value	Total (*n* = 57) *p*-Value	I (*n* = 13) *p*-Value	N-I (*n* = 44) *p*-Value	Total (*n* = 57) *p*-Value	I (*n* = 13) *p*-Value	N-I (*n* = 44) *p*-Value	Total (*n* = 57) *p*-Value	I (*n* = 13) *p*-Value	N-I (*n* = 44) *p*-Value	Total (*n* = 57) *p*-Value
TUG-T	0.214	0.071	0.005 *	0.187	0.644	0.525	0.853	0.675	0.649	0.175	0.385	0.840	0.472	0.374	0.557
TUG-R	0.730	0.002 *	0.002 *	0.337	0.287	0.429	-	0.044 *	0.002 *	0.054	0.559	0.184	0.554	0.161	0.012 *
30s Chair-T	0.884	0.258	0.037 *	1.00	0.709	0.210	0.556	0.988	0.901	0.293	0.981	0.451	00.674	0.177	0.414
30s Chair-R	0.842	0.171	0.171	0.30	0.450	0.317	-	0.536	0.044 *	0.823	0.704	0.676	0.866	0.250	0.026 *
SPPB-T	0.393	0.003 *	0.000 *	0.199	0.053	0.935	0.706	0.847	0.789	0.462	0.621	0.316	0.248	0.025 *	0.196
SPPB-R	0.103	0.018 *	0.018 *	0.104	0.938	0.169	-	0.095	0.005 *	0.343	0.707	0.544	0.659	0.990	0.124

30s Chair = chair stand test; I = Institutionalized; N-I = No- Institutionalized; R = re-test; SPPB = short physical performance battery; T = test; TUG = timed up and go; * *p*-value ≤ 0.05.

## Data Availability

The datasets generated and/or analyzed during the present study are not publicly available because participants consented to keep their information confidential. Nevertheless, the data may be made available from the corresponding authors upon reasonable request.

## Supplementary Materials

The following supporting information can be downloaded at: https://www.mdpi.com/article/10.3390/sports13110375/s1, Document S1: Five Low-Cost Tests to identify compensatory protective step strategies.
