# Supplementary material for: Evaluating the Test–Retest Reliability of Five Low-Cost, Perturbation-Based Functional Tests for Balance Recovery in Older Adults
Source: Sports (Basel). 2025 Nov 3;13(11):375. doi: 10.3390/sports13110375 (PMC12655952; doi:10.3390/sports13110375)
Supplement: Supplementary file 1 [file sports-13-00375-s001.zip › sports-3845303-supplementary.pdf]

Five Low-Cost Tests to identify compensatory protective step strategies

Objective: To identify protective compensatory stepping strategies used by older adults to prevent falls. During these tests, participants are exposed to different perturbations and must solve the motor challenge to avoid falling.

Relevance: Detecting protective compensatory stepping strategies is important because individuals who use multiple steps, crossover steps, and/or compensatory steps with limb collisions are at greater risk of falling.

Materials:

- 1 measuring tape.
- 1 A4 sheet of paper.
- 1 obstacle (40 cm long × 14 cm high × 8–9 cm wide).
- 1 non-elastic rope (1.5 m).

Time: 5–10 minutes.

Interpretation of possible outcomes: Lower scores indicate safer and more efficient strategies for resolving the motor challenge.

<u>Test Instructions</u>

1. Obstacle test

- **Evaluator considerations:** Place the obstacle at the midpoint of the 7 m walkway (at 3.5 m). Stand outside the walkway, on the side of the obstacle, to observe lower limb movements.
- **Participant instructions:** "*Stand on the floor mark (0 m) with your feet hip-width apart. Now, walk at your normal walking speed to the end of the walkway (7 m), looking straight ahead*."
- -**Strategy to evaluate**: Long step strategy.

1 Uses a long step strategy to clear the obstacle.

2 Uses a short step strategy to clear the obstacle.

3 Fails to use the chosen strategy effectively, trips over the obstacle, and interrupts the gait.

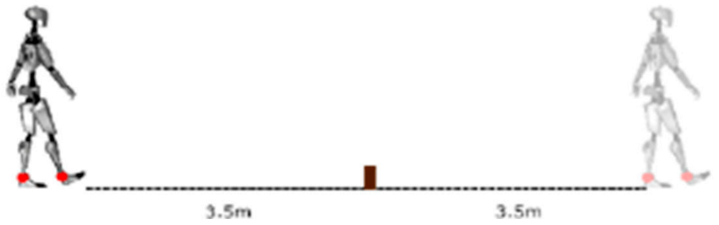

2. Push Forward Test

- **Evaluator considerations:** Tie the rope around the participant's waist (hip level). Place the free end of the rope along the participant's back, hanging between the hip and knees (it must not touch the floor). Keep it in place for the next "backward perturbation" test. Stand behind the participant and deliver a quick, strong, and sharp push to the upper back (keep your hand ~4 cm from the back), inducing a forward imbalance. WARNING: Be ready to catch the participant if necessary.
- **Participant instructions:** *"I will place this rope around your waist. Stand with your feet hip-width apart and arms relaxed. I will now create different imbalances. You must do whatever is necessary to avoid falling. Take as many steps as needed to recover your balance and remain still once stable."*
- **Strategy to evaluate:** Single forward step strategy.

1 Uses a single forward step strategy, longer than a normal step, to stabilize.

2 Uses multiple steps strategy to stabilize.

3. Pull Backward Test

- **Evaluator considerations:** Stand behind the participant. Hold an A4 sheet in one hand and the free end of the rope in the other. Place the A4 sheet horizontally at hip height (evaluator's hip). Pull the rope until it reaches the far edge of the sheet (relative to your body). Release the sheet and give a quick, sharp pull of the rope towards your hip to induce a backward imbalance. WARNING: Be ready to catch the participant if necessary.
- **Participant instructions:** *"Stand with your feet hip-width apart and arms relaxed. I will now create different imbalances. You must do whatever is necessary to avoid falling. Take as many steps as needed to recover your balance and remain still once stable."*
- **Strategy to evaluate:** Single backward step strategy.

1 Uses a single backward step strategy, longer than a normal step, to stabilize.

2 Uses multiple steps strategy to stabilize.

4. Pull Lateral Test

- **Evaluator considerations:** Stand facing the participant's side. Position the rope so its free end is at the hip level of the side facing you. Hold an A4 sheet in one hand and the free end of the rope in the other. Place the A4 sheet horizontally at your hip height. Pull the rope until it reaches the far edge of the sheet (relative to your body). Release the sheet and give a quick, sharp pull of the rope towards your hip to induce a lateral imbalance. WARNING: Be ready to catch the participant if necessary.
- **Participant instructions:** *"Stand with your feet hip-width apart and arms relaxed. I will now create different imbalances. You must do whatever is necessary to avoid falling. Take as many steps as needed to recover your balance and remain still once stable."*
- **Strategy to evaluate:** Loaded lateral step.

Pull from the participant's left side:

1 Uses a loaded sidestep strategy to stabilize.

2 Uses an unloaded sidestep strategy to stabilize.

2 Uses a medial sidestep strategy to stabilize.

3 Uses a crossover step strategy to stabilize.

4 Limb collision compensatory step strategy.

Pull from the participant's right side:

1 Uses a loaded sidestep strategy to stabilize.

2 Uses an unloaded sidestep strategy to stabilize.

2 Uses a medial sidestep strategy to stabilize.

3 Uses a crossover step strategy to stabilize.

4 Limb collision compensatory step strategy.
